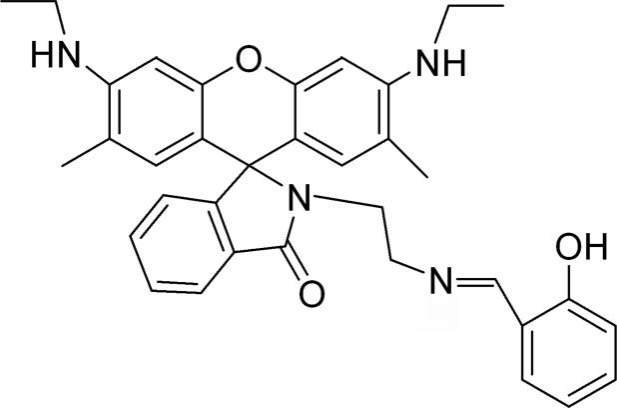# [(2-{[3′,6′-Bis(ethyl­amino)-2′,7′-dimethyl-3-oxospiro­[1*H*-isoindole-1,9′-9*H*-xanthen]-2-yl}eth­yl)amino­meth­yl]phenol. Corrigendum

**DOI:** 10.1107/S1600536808011859

**Published:** 2008-05-14

**Authors:** Li-Zhu Zhang, Xiao-Jun Peng, Shang Gao, Jiang-Li Fan

**Affiliations:** a State Key Laboratory of Fine Chemicals, Dalian University of Technology, 158 Zhongshan Road, Dalian 116012, People’s Republic of China

## Abstract

The title and the chemical diagram of the paper by Zhang, Peng, Gao & Fan [*Acta Cryst.* (2008), E**64**, o403] are corrected.

## Abstract 2

Corrigendum to *Acta Cryst.* (2008), E**64**, o403.

In the paper by Zhang, Peng, Gao & Fan [*Acta Cryst.* (2008), E**64**, o403], the title and the chemical diagram are incorrect. The correct structure is shown below and the correct title of the original paper should be ‘[(2-{[3′,6′-Bis(ethyl­amino)-2′,7′-dimethyl-3-oxospiro­[1*H*-isoindole-1,9′-9*H*-xanthen]-2-yl}eth­yl)imino­meth­yl]phenol’.